# Circulating Surfactant Protein D: A Biomarker for Acute Lung Injury?

**DOI:** 10.3390/biomedicines11092517

**Published:** 2023-09-12

**Authors:** Alyssa Elmore, Ali Almuntashiri, Xiaoyun Wang, Sultan Almuntashiri, Duo Zhang

**Affiliations:** 1 College of Pharmacy, University of Georgia, Augusta, GA 30912, USA; 2 Department of Dentistry, Security Forces Hospital, Dammam 32314, Saudi Arabia; 3 Department of Preventive Dentistry, College of Dentistry, Qassim University, Ar Rass 52571, Saudi Arabia; 4 Clinical and Experimental Therapeutics, College of Pharmacy, University of Georgia and Charlie Norwood VA Medical Center, Augusta, GA 30912, USAduozhang@uga.edu (D.Z.); 5 Department of Clinical Pharmacy, College of Pharmacy, University of Hail, Hail 55473, Saudi Arabia

**Keywords:** ALI, ARDS, biomarker, lung injury, lung inflammation

## Abstract

Acute lung injury (ALI) and acute respiratory distress syndrome (ARDS) are life-threatening lung diseases in critically ill patients. The lack of prognostic biomarkers has halted detection methods and effective therapy development. Quantitative biomarker-based approaches in the systemic circulation have been proposed as a means of enhancing diagnostic strategies as well as pharmacotherapy in a patient-specific manner. Pulmonary surfactants are complex mixtures made up of lipids and proteins, which are secreted into the alveolar space by epithelial type II cells under normal and pathological conditions. In this review, we summarize the current knowledge of SP-D in lung injury from both preclinical and clinical studies. Among surfactant proteins, surfactant protein-D (SP-D) has been more widely studied in ALI and ARDS. Recent studies have reported that SP-D has a superior discriminatory ability compared to other lung epithelial proteins for the diagnosis of ARDS, which could reflect the severity of lung injury. Furthermore, we shed light on recombinant SP-D treatment and its benefits as a potential drug for ALI, and we encourage further studies to translate SP-D into clinical use for diagnosis and treatment.

## 1. Introduction

Acute lung injury (ALI) and acute respiratory distress syndrome (ARDS) are life-threatening pulmonary diseases in critically ill patients, affecting around 200,000 patients every year in the United States and approximately 10% of all patients during intensive care unit (ICU) admissions [1,2,3,4,5]. These threatening diseases are characterized by exudative alveolar flooding and extensive alveolar collapse due to the disruption of the air–blood barrier and surfactant abnormalities, respectively [6,7]. Despite notable advances in the diagnosis and treatment of ARDS over the last 50 years, the risk of mortality remains high at 30–40% [8]. The lack of prognostic biomarkers has halted detection methods and effective therapy development [9]. Quantitative biomarker-based approaches in systemic circulation have been proposed as a means of enhancing diagnostic strategies as well as pharmacotherapy in a patient-specific manner [6].

Pulmonary surfactants are a complex mixture of lipids and proteins that are secreted into the alveolar space by epithelial type II cells [10]. The main role of surfactants is to diminish surface tension in the alveoli at the air–liquid interface and inhibit its collapse at end-expiration [11]. Currently, there are four known surfactant proteins encoded in the human genome—surfactant protein (SP) A, B, C, and D. These proteins are divided into hydrophilic and hydrophobic groups. SP-A and -D are hydrophilic proteins known for their roles in pulmonary immunity and the regulation of inflammation. The molecular weight of SP-A is 36 kDa, while the molecular weight of SP-D is 43 kDa. Both SP-A and SP-D are homologous in their sequences [12]. SP-B and -C are hydrophobic proteins that play essential roles in the normal function of pulmonary surfactants. Their biological activities include maintaining surfactant structures and stabilizing lipid layers over respiratory cycles [13]. SP-B is the smallest surfactant, with an 8 kDa molecular weight in its mature form, and SP-C has a 21 kDa molecular weight [14,15] (Table 1). Previous studies have shown that club cells can synthesize and secrete mature surfactants SP-A, -B, and -D [16,17]. In addition, type II alveolar epithelial cells are known to synthesize and release all surfactant components, including surfactant proteins and phospholipids [18,19,20].

SP-D is one of the collectin proteins found in the lungs. SP-D plays a crucial role in the innate immune response by recognizing harmful particles and promoting the phagocytosis process [21]. Moreover, SP-D regulates inflammatory responses by modulating inflammatory pathways such as the toll-like receptor 4 (TLR4) signaling pathway [22]. SP-D also influences lung surfactant lipid homeostasis and alveolar structures [23]. In regard to its structure, SP-D consists of four distinct structural domains: N-terminal domain, collagenous domain, coiled-coil neck domain, and C-type lectin carbohydrate recognition domain (CRD) [24]. It is known that SP-D is secreted by type II pneumocytes in different oligomeric forms, including trimers, hexamers, and dodecamers. Dodecamers and higher-order oligomers are the most active oligomeric forms for the lectin-mediated functions of SP-D [23,25].

Lung injury and inflammation influence the secretion of surfactant proteins from lung epithelial cells into the blood circulation. The detection of such proteins in the plasma or serum at high concentrations could reflect an abnormality in the pulmonary epithelial barrier/air-blood barrier [11]. Therefore, they have been widely examined as circulating diagnostic and/or prognostic biomarkers for lung diseases, such as ARDS, chronic obstructive pulmonary disease (COPD), and idiopathic pulmonary fibrosis (IPF) [26,27,28,29].

In this review, we thoroughly summarize the current results for SP-D in ALI/ARDS from preclinical and clinical studies. Our review focuses on SP-D since it was recently shown that SP-D had a better discriminatory ability for the diagnosis of ARDS than other lung epithelial proteins [30]. Furthermore, we shed light on recombinant SP-D, which has been investigated as a potential drug therapy for ALI.

## 2. SP-D in Clinical Studies

**Figure 1:**
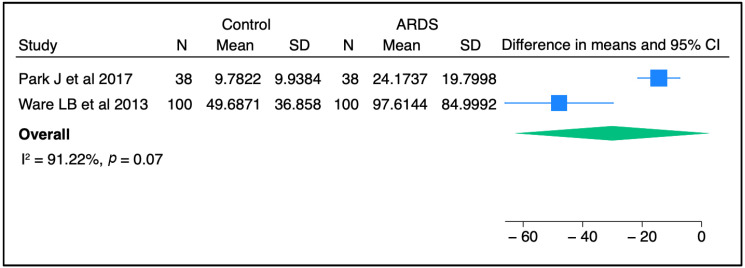
SP-D concentrations between control and ARDS subjects from two independent studies [30,31]. Meta-analysis was performed using STATA, version 18. Means and standard deviations were calculated using the methods of Luo et al. [37] and Wan et al. [38], respectively.

Circulating SP-D has been investigated as a potential prognostic and/or diagnostic biomarker for ALI/ARDS over recent decades. In most previous studies, the plasma level of SP-D was significantly higher in ARDS patients than in controls, raising the possibility for SP-D to serve as a non-invasive diagnostic biomarker for lung injury [30,31,32]. Upon meta-analysis of two independent studies, there was a trend toward a higher concentration of SP-D in ARDS patients than in non-ARDS controls (Figure 1). Furthermore, serum SP-D concentrations were significantly higher in direct ARDS patients when compared to other ARDS patients caused by extrapulmonary origins in a study conducted by Zhonghua and colleagues [33]. Likewise, Calfee et al. found that plasma SP-D levels were significantly higher in cases with direct ARDS than indirect ARDS [34]. In line with these findings, Park J et al. stated that SP-D levels were slightly higher in patients with direct lung injury compared to those with indirect lung injury, but this change did not reach a statistically significant level [31]. The sample population in the latter study was smaller than that in the other two clinical studies, which could explain the non-significant findings. Interestingly, a recent study reported that circulating SP-D levels were significantly altered in response to a causative pathogen. The authors found that SP-D was significantly higher in direct ARDS caused by viral and atypical pathogens than in ARDS caused by typical bacterial pneumonia [35]. Another study suggested that SP-D could be a good predictive biomarker for poor outcomes in ARDS caused by viral pneumonia [36]. Overall, it appears that SP-D in the circulation might serve as a diagnostic biomarker for lung injury and has the potential to differentiate between direct/indirect lung injury, and reflect certain causative pathogens.

**Figure 2:**
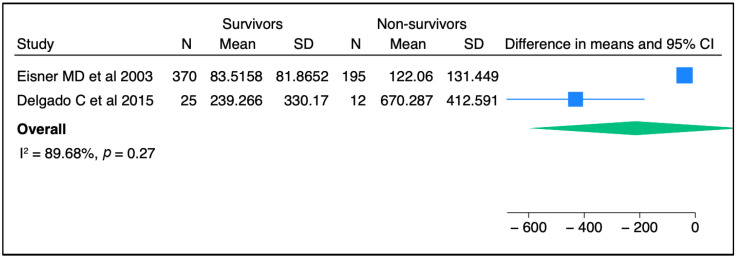
The comparison of SP-D concentrations between survivors and non-survivors among ARDS patients from two independent studies [36,43]. Meta-analysis was performed using STATA, version 18. Means and standard deviations were calculated using the methods of Luo et al. [37] and Wan et al. [38], respectively.

**Figure 3:**
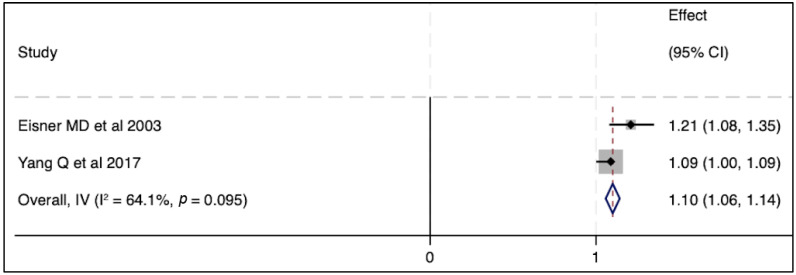
The effect of elevated SP-D concentrations on mortality outcome [33,43]. Pooled odds ratios were calculated using STATA, version 18.

In terms of severity of ARDS, there were no associations between circulating SP-D levels and lung injury severity parameters in two published studies [39,40], whereas another two studies reported a significant correlation between SP-D and lung injury score [41,42] (Table 2). Regarding mortality outcomes, elevated SP-D plasma levels were significantly associated with the risk of death in multiple cohort studies. Three independent studies reported higher circulating SP-D levels in non-survivors when compared to those in live patients [36,41,43]. Likewise, an elevated baseline circulating SP-D level was associated with a greater mortality risk and worse clinical outcomes [43]. Similarly, an elevated serum level of SP-D was an independent prognostic factor for the risk of mortality in another cohort study [33]. In contrast to these aforementioned studies, Greene KE et al., in one single cohort, stated that the circulating SP-D level was neither a specific nor sensitive predictor of mortality after the progression of ARDS [42]. When we conducted a meta-analysis of two independent studies, no significant association was seen between SP-D and mortality (Figure 2). Likewise, elevated SP-D was not significantly related to mortality in pooled odds ratios from two studies (Figure 3). In short, the SP-D concentration in the blood appeared to be a prognostic predictor of worse outcomes, including risk of death, in most previous clinical reports but did not appear as such in our meta-analysis.

Pediatric acute respiratory distress syndrome (PARDS) is one of the most critical conditions managed in pediatric intensive care units (PICUs). PARDS is characterized by the progression of consecutive inflammatory events that eventually disrupt the alveoli–capillary membrane. The pathophysiology of PARDS is complex since it involves a causative nature and body–host responses [49]. The current diagnosis for PARDS requires an assessment of several variables, such as physical exams, chest X-rays, and oxygenation indices [50]. Circulating biomarkers have been studied in PARDS due to their potential benefits in diagnosis, prognostication, and the assessment of therapeutic responses [51]. In particular, SP-D has been investigated as a useful diagnostic and prognostic blood marker for PARDS in clinical settings. For instance, Chakrabarti et al. reported that SP-D in circulation was significantly correlated with the severity of PARDS, mechanical ventilation, ICU, hospital length of stay, and Pediatric Risk of Mortality (PRISM III) scores in influenza-infected children [44]. Likewise, Dahmer et al. showed that SP-D concentrations were significantly associated with severity of PARDS, duration of mechanical ventilation, PICU length of stay, and risk of death [45]. In relation to oxygen indices, two independent studies showed that elevated plasma SP-D was significantly associated with poor oxygenation index (OI) [45,47]. Overall, circulating SP-D measurements were associated with the severity of clinical parameters and poor outcomes, including death in children, supporting the application of circulating SP-D as a prognostic marker in PARDS.

## 3. SP-D in Preclinical Studies

Current in vivo studies suggest that SP-D plays a role in lung inflammation and can reflect the severity of lung injury when measured from the circulation. These preclinical studies utilized various treatments to induce lung injury in animals. The treatments included were malaria-induced ALI, lipopolysaccharide (LPS), influenza, *Pneumocystis carinii,* bleomycin, and pathogenic bacteria, such as *Staphylococcus aureus* and *Pseudomonas aeruginosa* (Table 3). In a mouse model with malaria-induced ALI, the amount of SP-D protein found in the lungs was vastly elevated when compared to that in control groups [52]. Continuously increased serum SP-D levels were observed in response to chronic lung injury (*Pneumocystis carinii*) when compared to acute lung injury induced by LPS or bleomycin [53]. In acute injury models, serum SP-D levels were also significantly elevated on day 5, peaked on day 10, and gradually decreased until day 28 after bleomycin administration [54]. In the latter study, conventional biomarkers, such as lactate dehydrogenase (LDH), monocyte chemoattractant protein-1 (MCP-1), and C-reactive protein (CRP), were only elevated for a few days in the serum, and did not change significantly over time. In the same study, SP-D levels in bronchoalveolar lavage fluid (BALF) increased and peaked on day 3, and significantly correlated with total cell counts, granulocyte cell counts, serum albumin levels, and the wet lung weight/body weight ratio [54]. Overall, studies indicate that circulating SP-D reflects some pathological alterations in the lungs and is a useful prognostic tool for lung injury and its complications in vivo.

It is well recognized that the SP-D protein is largely secreted from alveolar epithelial cells and, to some extent, from other pulmonary cells, such as non-ciliated bronchial epithelial cells or club cells. For instance, cytoplasmic positive staining of SP-D was found in alveolar epithelial cells, mainly alveolar type II cells, and alveolar macrophages in malaria-infected mice [52]. Likewise, strong immunoreactivity for SP-D was seen on macrophages and type II pneumocytes upon LPS exposure, and SP-D appeared to translocate from airways into the vascular system [53]. Similarly, SP-D immunoreactivity was seen on type II pneumocytes, club cells, and alveolar macrophages, while elevated SP-D expression was noted in alveolar type II cell hyperplasia from day 3 to day 10 after bleomycin treatment [54]. These findings confirmed that SP-D is produced from lung cells, mainly alveolar type II cells, in response to lung injury and can be easily detected in the circulation. This supports the clinical application of SP-D as a diagnostic or prognostic biomarker for ALI.

Loss-of-function and gain-of-function strategies have been applied to preclinical research for decades, and their positive impacts are undoubtable. Previously, three independent studies employed the loss-of-function strategy to evaluate the role of SP-D in response to lung injury by using SP-D KO mice, and one study employed the gain-of-function strategy by using SP-D overexpressing mice. SP-D KO mice did not show a higher mortality than WT mice after LPS treatment as reported by King et al. [55]. In contrast, after receiving bleomycin, a higher mortality rate was seen in SP-D KO mice compared to WT mice [56]. Likewise, a higher mortality rate was observed in KO mice than in WT after *Pseudomonas aeruginosa* infection [58]. In a study of mice overexpressing SP-D, the authors reported that mice were significantly resistant to bleomycin-induced lung injury, as reflected by reduced severity and mortality [56]. In conclusion, SP-D plays an essential and protective role against lung injury, whereas loss of SP-D may deteriorate lung conditions after pathogenic or sterile stimulus.

In SP-D KO mice, lung levels of inflammatory mediators, such as IL-6 and TNF-α, were higher than in WT mice. Additionally, the levels of inflammatory mediators were higher in the lung after indirect than direct lung injury when performed by intraperitoneal and intratracheal injections of LPS, respectively. In the same study, the number of macrophage-specific antibody (MAC-3)-positive cells and levels of granulocyte-macrophage colony-stimulating factor (GM-CSF) increased significantly in SP-D KO mice after indirect lung injury. The authors concluded that after such injury, SP-D inhibited lung inflammation and migration of peripheral immune cells into the lung through GM-CSF-dependent pathways [55]. Moreover, another study showed that more severe pulmonary parenchymal inflammation and BAL cellularity were seen in SP-D KO mice after bleomycin exposure [56]. Likewise, the number of neutrophils and macrophages in the BALF was higher in KO mice than in WT after *Pseudomonas aeruginosa* administration [58]. In the last two studies, SP-D KO mice had greater injury scores than WT [56,58]. Altogether, these studies confirmed the protective role of SP-D in lung injury by inhibiting cytokine/chemokine induction and immune cell migration into the lung.

## 4. Recombinant SP-D and Its Potential Benefits as a Treatment for ALI

In addition to its possible role as a circulating biomarker for lung injury, SP-D has also been investigated as a potential drug therapy for acute and chronic pulmonary diseases, including COPD, influenza A virus, coronavirus disease 2019 (COVID-19), and ALI. In a study of COPD, SP-D inhibited lipid-laden foamy macrophages (FMs), which are frequently seen under oxidative stress and have defective phagocytic functions. This study supported the essential role of SP-D in the lipid stability of alveoli and provided a possibility for the clinical application of SP-D as a treatment for lung inflammation and COPD [59]. Another study showed that recombinant SP-D inhibited influenza A virus replication, suggesting that SP-D could be utilized as a platform to develop a potential class of antiviral drugs [60]. Likewise, recombinant SP-D downregulated mRNA levels of pro-inflammatory mediators in vitro during the early stage of influenza A virus infection, indicating that SP-D diminished aberrant lung inflammation and lung damage induced by influenza infections [61]. Moreover, SP-D inhibited severe acute respiratory syndrome coronavirus 2 (SARS-CoV-2) replication in vitro, an enveloped RNA virus responsible for the COVID-19 pandemic, denoting the possibility of SP-D as a drug therapy in immune responses triggered by SARS-CoV-2 infection [62]. Consistently, recombinant SP-D also inhibited SARS-CoV-2 replication after binding to the SARS-CoV-2 Spike protein and prevented viral entry into cells expressing angiotensin-converting enzyme 2 (ACE2), an important receptor for cell entry of SARS-CoV-*2*. In the same study, recombinant SP-D significantly downregulated the mRNA levels of pro-inflammatory mediators like TNF-α, interferon-alpha (IFN-α), interleukin-1β (IL-1β), IL-6, IL-8, and regulated upon activation normal T cell expressed and secreted (RANTES) in vitro, suggesting an additional protective role for recombinant SP-D in SARS-CoV-2 infections [63]. In lung injury models induced by LPS and lipoteichoic acid (LTA) treatments, intratracheal recombinant SP-D prevented lung inflammation manifestations, including neutrophilic infiltrates [64]. In a model of ventilation-induced inflammation and lung injury, recombinant SP-D also diminished neutrophil counts and neutrophil elastase activity in BALF and lung tissue, respectively [65]. Overall, these studies supported the potential use of recombinant SP-D as a treatment for patients with lung injury.

## 5. Challenges and Future Directions

Despite the major consistent findings for SP-D as a potential non-invasive biomarker for ALI, there are still some inconsistent data, particularly when we compare preclinical findings to clinical findings. For instance, most clinical reports showed a greater concentration of SP-D in patients with direct lung injury whereas the opposite was observed in preclinical studies. Moreover, although SP-D concentrations were obviously altered in response to lung injury, big variations were observed from previous reports and a reference value is still missing. In addition, several factors may affect SP-D concentration and need to be taken into consideration, like age [45] and weight [46]. Furthermore, the molecular weight of SP-D is higher than other surfactant proteins, and this could affect its stability in the systemic circulation, and also after collection for laboratory measurements. Lastly, treatment with recombinant SP-D may alleviate ARDS severity in humans based on the current literature, but there is a lack of preclinical research aimed primarily at assessing the efficacy and safety of SP-D. This is an essential step for developing recombinant SP-D as a drug therapy in clinical settings. In short, more studies are still needed for the development of SP-D as a potential biomarker and a therapeutic agent for patients with ALI.

## 6. Conclusions

In conclusion, the current review has focused on SP-D as a potential diagnostic and prognostic biomarker for lung injury by summarizing available preclinical and clinical studies. The current findings indicate that SP-D is translocated from the alveoli into the systemic circulation, suggesting that SP-D can non-invasively reflect the severity of lung injury. Nevertheless, extensive validation is necessary to establish the clinical usefulness of SP-D as a potential biomarker for lung injury. Moreover, this review has shed light on recombinant SP-D as a potential drug therapy for pulmonary diseases, particularly ALI and ARDS.

## Figures and Tables

**Table 1:** Surfactant protein categorization.

Surfactant Protein	Solubility	Molecular Weight	Predominant Structure	Biological Roles in the Lung
SP-A	Hydrophilic	36 kDa	Hexamer	- Reduce the surface tension - Regulate immune response
SP-B	Hydrophobic	8 kDa	Homodimer	- Essential for normal lung surfactant function - Maintain physiological surface properties
SP-C	Hydrophobic	21 kDa	Monomer	- Stabilize the alveolar surfactant film - Lower the surface tension
SP-D	Hydrophilic	43 kDa	Dodecamer	- Regulate pulmonary surfactants and lipid homeostasis - Serve as a lung host defense protein

**Table 2:** The change in circulating SP-D in ALI/ARDS patients and its association with severity and mortality outcomes.

Main Findings	Reference
Higher SP-D levels were significantly associated with fewer ventilator-free days	[43]
Higher SP-D levels were significantly associated with fewer organ-failure-free days	
SP-D was significantly associated with the number of days on a ventilator and length of stay in hospital	[41]
The baseline correlation of SP-D with lung injury score was significant	
SP-D was significantly correlated with the lung injury score on day 3 of ARDS	[42]
Circulating SP-D was neither a specific nor sensitive predictor of mortality after the progression of ARDS	
Higher levels of SP-D were significantly associated with fatal outcomes in ARDS patients caused by influenza A virus (H1N1)	[36]
Circulating SP-D was significantly correlated with a diagnosis of moderate to severe PARDS, mechanical ventilator, ICU, and hospital length of stay in influenza-infected children	[44]
Plasma SP-D peaked on day 4 and remained elevated until day 9	
SP-D significantly correlated with PRISM III score	
Multivariable regression analyses indicated that elevated SP-D concentrations were significantly associated with risk of death, duration of mechanical ventilation, pediatric ICU length of stay, and higher oxygenation index	[45]
SP-D was significantly higher in non-survivors than in living children	
SP-D concentrations were significantly and positively correlated with age	
Obesity was significantly associated with lower circulating SP-D levels among children with PARDS	[46]
Elevated plasma SP-D was significantly associated with oxygenation index (OI) and lung injury severity (LIS) on day 1 among PARDS patients	[47]
Direct lung injury led to higher SP-D levels in children than indirect lung injury	
There was a significant association between day 1 SP-D levels and in-hospital mortality	
The serum concentrations of SP-D were significantly increased in ARDS patients with pulmonary superinfections in comparison to ARDS patients without pulmonary superinfections	[48]

Abbreviations: ALI, acute lung injury; ARDS, acute respiratory distress syndrome; PARDS, pediatric acute respiratory distress syndrome; SP-D, surfactant protein D; ICU, intensive care unit; PRISM III, pediatric risk of mortality; OI, oxygenation index; LIS, lung injury severity.

**Table 3:** Main findings for SP-D in preclinical models of lung injury.

Reference	Experimental Model	Conclusion	Change in SP-D
[55]	LPS induced ALI	Lung levels of interleukin 6 (IL-6) and tumor necrosis factor alpha (TNF-α) were higher in SP-D knockout (KO) mice than in wild-type (WT) mice and were elevated more after indirect than direct lung injury.	-
		The number of macrophage-specific antibody (MAC-3)-positive cells increased approximately 2-fold in SP-D KO mice after indirect lung injury.	-
		The level of granulocyte-macrophage colony-stimulating factor (GM-CSF) was approximately 5-fold greater in SP-D KO mice than in WT mice in response to indirect injury.	-
[53]	LPS, bleomycin, or Pneumocystis carinii induced ALI	Serum SP-D levels increased continuously during chronic lung injury compared to acute injury.	↑
		Strong immunoreactivity for SP-D was seen on macrophages and type II pneumocytes upon LPS exposure.	-
		SP-D translocated from the airways into the vascular system.	-
[54]	Bleomycin induced ALI	Serum SP-D levels reflected pathological alterations in the lungs, and the measurement of SP-D was a useful tool for detecting lung injury.	↑
		SP-D immunoreactivity was seen on type II pneumocytes, Club cells, and alveolar macrophages.	-
		Elevated SP-D expression was noted in alveolar type II cell hyperplasia on day 3.	-
		SP-D levels in BALF increased and peaked on day 3 and significantly correlated with the total cell count, the granulocyte cell count, the serum albumin level, and the wet lung weight/body weight ratio.	↑
		SP-D levels in serum were significantly elevated on day 5 and peaked on day 10.	↑
[56]	Bleomycin induced ALI	Higher mortality, greater respiratory distress, and weight loss were seen in SP-D KO mice after receiving bleomycin compared to WT.	-
		More pulmonary parenchymal inflammation and BAL cellularity were seen in SP-D KO mice.	-
		More trichrome staining and higher hydroxyproline levels were noted in the lung tissues of SP-D KO mice.	-
		SP-D-overexpressing mice were significantly resistant to bleomycin-induced severity and mortality.	-
[57]	*Staphylococcus aureus* induced ALI	More severe infection was seen in double knockout (SP-A/D KO) mice than in WT controls.	-
[58]	*Pseudomonas aeruginosa* induced ALI	SP-D KO mice showed greater injury scores in the lung than WT.	-
		A higher mortality rate was observed in infected KO mice compared to infected WT.	-
		BALF neutrophil and macrophage counts were higher in KO mice than in WT after infection.	-
		SP-D concentrations in the lung were reduced at 48 h after infection in WT.	↓
[44]	Influenza induced ALI	Circulating SP-D showed a greater increase in mice following influenza infection than other proteins.	↑
[52]	Malaria induced ALI	Malaria-infected mice had an increased protein level of SP-D in the plasma and lungs compared to uninfected mice.	↑
		A significant positive correlation was seen between SP-D in the plasma and the lung.	↑
		Positive staining of SP-D was found in alveolar type II cells and alveolar macrophages.	-

Abbreviations: ALI, acute lung injury; ARDS, acute respiratory distress syndrome; SP-D, surfactant protein D; IL-6, interleukin 6; TNF-α, tumor necrosis factor-alpha; KO, knockout; WT, wild-type; MAC-3, macrophage-specific antibody; GM-CSF, granulocyte-macrophage colony-stimulating factor; BALF, bronchoalveolar lavage fluid; ↑, increase; ↓, decrease.
